# Induction of the mitochondria-mediated apoptosis in human esophageal cancer cells by DS2, a newly synthetic diterpenoid analog, is regulated by Bax and caused by generation of reactive oxygen species

**DOI:** 10.18632/oncotarget.13367

**Published:** 2016-11-11

**Authors:** Yong-Cheng Ma, Yu Ke, Xiaolin Zi, Fei Zhao, Lin Yuan, Ying-Li Zhu, Xia-Xia Fan, Ning-Min Zhao, Qiao-Yan Li, Yu-Hua Qin, Hong-Min Liu

**Affiliations:** ^1^ Clinical Pharmacology Laboratory, Zhengzhou University People's Hospital, Zhengzhou, Henan, China; ^2^ School of Pharmaceutical Sciences and Collaborative Innovation Center of New Drug Research and Safety Evaluation, Zhengzhou University, Zhengzhou, China; ^3^ Department of Urology, University of California, Irvine, California, USA; ^4^ Department of Pharmacology, University of California, Irvine, California, USA; ^5^ Chao Family Comprehensive Cancer Center, University of California, Irvine, California, USA

**Keywords:** ent-kaurene diterpenoid derivative, esophageal squamous cell carcinoma, ROS, mitochondrion, Bax

## Abstract

Ent-kaurane diterpene compounds have attracted considerable attention in recent years due to its antitumor, antibacterial, and antiviral activities. However, the clinical development of natural kaurane diterpenes, for example, oridonin for cancer therapy has been hampered by its relatively moderate potency, limited bioavailability. Herein, we report a newly synthetic analog of natural ent-kaurane diterpene, DS2, which exhibits significantly improved activity of antiproliferation against various cancer cell lines relative to oridonin. DS2 treatment triggers the mitochondria-mediated apoptosis and cell cycle arrest in human esophageal cancer cell lines (EC9706, EC109). Interestingly, normal human esophageal epithelial cells (HEECs) and normal human liver cells (HL-7702) are both significantly more resistant to the growth inhibition by DS2 compared with esophageal cancer cells. The DS2-induced apoptosis in EC9706 cells correlated with the drop of mitochondrial membrane potential (MMP), release of cytochrome c into the cytosol and activation of caspase-9 and -3. The induction of proapoptotic proteins p21 and Bax were also observed in DS2-treated cells. The DS2-induced apoptosis was significantly attenuated by knockdown of Bax proteins. Meanwhile, the DS2 treatment caused generation of reactive oxygen species (ROS) in human esophageal cancer cells, but not in HEECs, which was attenuated by pretreatment with ROS scavenger N-acetylcysteine (NAC). More interestingly, the antioxidants pretreatment completely attenuated DS2 mediated loss of the MMP and apoptosis, as well as Bax expression and growth inhibition. In conclusion, the present study reveals that the mitochondria-mediated cell death by DS2 is associated with Bax regulation and ROS generation, and understanding the function and mechanism of DS2 will help us to design better anti-cancer drugs.

## INTRODUCTION

Esophageal squamous cell carcinoma (ESCC) is esophageal cancer's dominate pathologic subtype with remarkable geographic variation in incidence. Eastern Asia demonstrates a comparatively high morbidity in comparison with western nations in the world [1]. For example, over 50% of all ESCC cases reported worldwide occurred in China [2]. Despite tremendous advances in diagnostic techniques and therapeutic methods, the prognosis for patients with ESCC continues to be unfavorable on account of deferred diagnosis and the inadequate efficacy of conventional therapy [3]. Therefore, it is presently pressing to develop novel agents for ESCC patients.

Natural products tend to be a source of inspiration for synthetic chemists attempting to acquire new molecular entities with distinct pharmacological activity [4, 5]. *Isodon rubescens* (“Donglingcao” in Chinese) is a significant source of a traditional Chinese herbal medicine that has been widely used for esophageal and cardia cancer's treatment for many years in China [6, 7]. Many ent-kaurane diterpenoids were isolated from this herb, such as Oridonin [8], Jaridonin [9] and Eriocalyxin B [10]. In recent years, a rising attention has been being attracted by ent-kaurane diterpenoids due to their unique and extensive pharmacological activities, especially anticancer activity. In China, the injection of oridonin was used alone or in combination with other chemotherapy drugs for liver cancer's treatment [11]. Increasing studies have also illustrated that oridonin exerts broad anti-tumor activities through regulating the cell cycle [12, 13], apoptosis [14, 15], and autophagy [16, 17], as well as the inhibition of migration and invasion [18]. Recently, from *Isodon rubescens*, another new ent-kaurene diterpenoid was isolated by us, named Jaridonin. Importantly, our previous results have shown that Jaridonin was more potent than oridonin in inhibiting proliferation and pro-apoptotic in various human cancer cell lines, such as colon cancer, gastric cancer and esophageal cancer cell lines [9, 19]. We were intrigued by Jaridonin's anticancer profile to make use of its special scaffold as an introductory template to synthesize novel Jaridonin derivatives to develop effective and safe anticancer agents. Numerous studies have identified sulfur-containing compounds possess multiple biological effects supporting their potential use in multi-targeted cancer prevention and treatment [20]. And recently, expeditious synthetic methods that were based on the Jaridonin scaffold were successfully built by our group to access a series of disulfide bond-substituted derivatives with improved anticancer activity, among them, DS2 (the synthesis and the structure identification of DS2 are shown in Supplementary Figures S1, S2 and S3) suggesting that disulfide bond-substituted modifications seem to be tolerated for producing biologically interesting molecules.

In the present study, we report the effect and potential mechanisms of DS2 in cancer cells. The results indicate that DS2's activity in ESCC cells involves the Bax regulation, and ROS generation and the activation of the mitochondria-mediated apoptotic pathway. It guarantees further research as an expected therapeutic agent for ESCC's treatment, and will increase our knowledge about the disulfide bond-substituted derivatives.

## RESULTS

### The anti-proliferation effects of DS2 in human cancer cells

**Figure 1:**
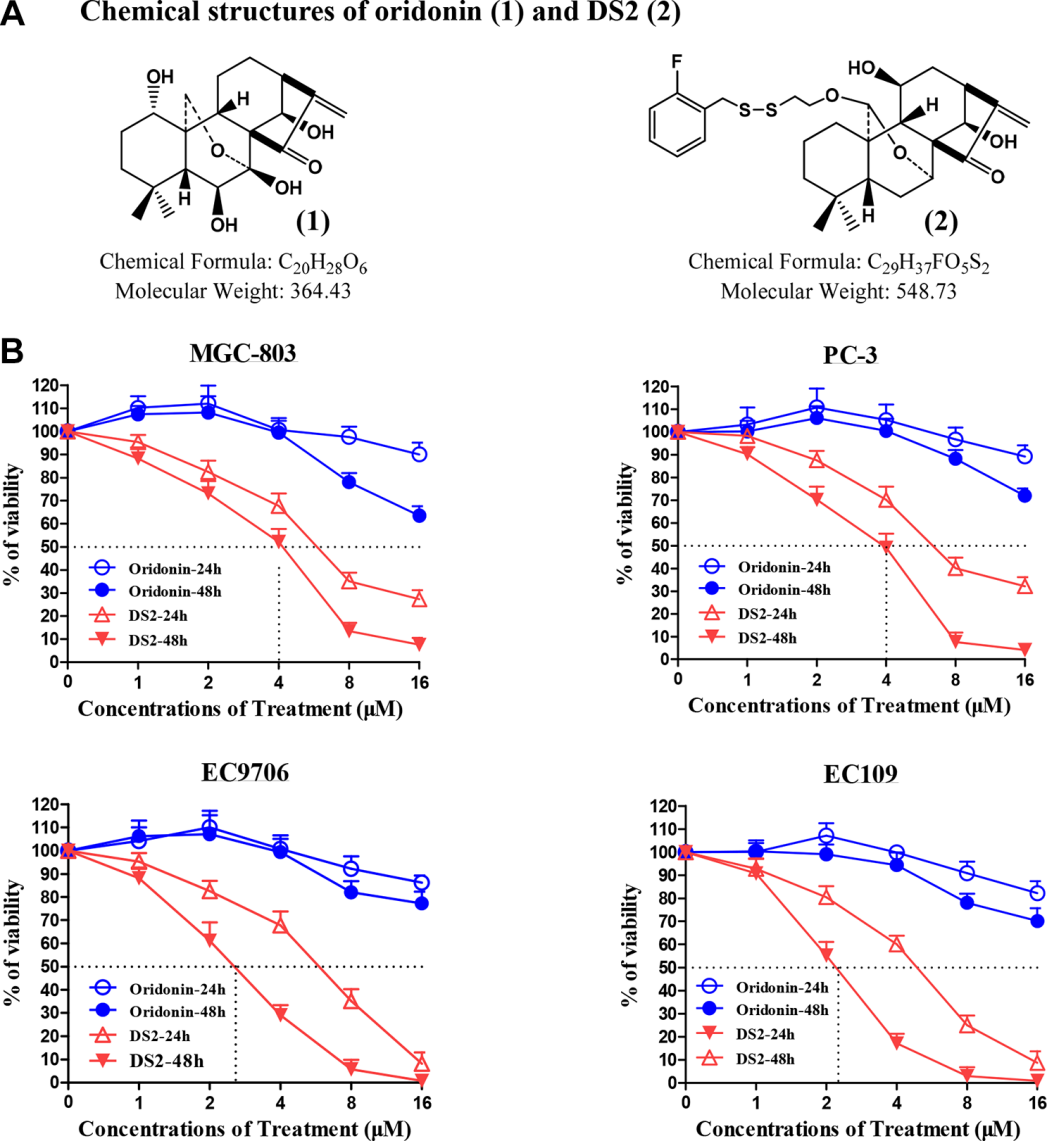
Effects of DS2 on the growth of human cancer cells (**A**) Chemical structures of oridonin (1) and DS2 (2). (**B**) 6 × 10^3^ MGC-803, PC-3, EC9706 and EC109 cells were plated in 96-well culture plates. 24 hours later, the cells were changed with fresh medium and treated with DS2 or oridonin at the indicated doses for 24 hours or 48 hours, 0.1% DMSO was used as controls. Cell viability was measured by MTT assay. Data are mean ± SD (*n* = 3).

Chemical structures of DS2 and oridonin, a representative ent-kaurane diterpernoid from *Isodon rubescens*, are shown as Figure 1A. Figure 1B shows that DS2 inhibits the growth of four human tumor cell lines, including gastric carcinoma cell MGC-803, prostate cancer cell PC-3, as well as ESCC cell lines EC9706 and EC109 in a time- and dose-dependent manner. Compared to control treatment, DS2 treatment at 4 μM concentration for 24 h or 48 h resulted in about 33% or 48%, 30% or 50%, 32% or 70% and 40% or 80% growth inhibition of MGC-803, PC-3, EC9706 and EC109 cells. However, these inhibitions were not observed on proliferation of all four cell lines, with 4 μM oridonin treatment even for 48 h. It appears that DS2 is more potent in inhibiting the growth of human cancer cells than oridonin. Since EC9706 and EC109 cells, in particular, were relatively sensitive to DS2, the subsequent studies were performed on the two cell lines.

### DS2 resulted in G2/M phase cell cycle arrest in ESCC cells

**Figure 2:**
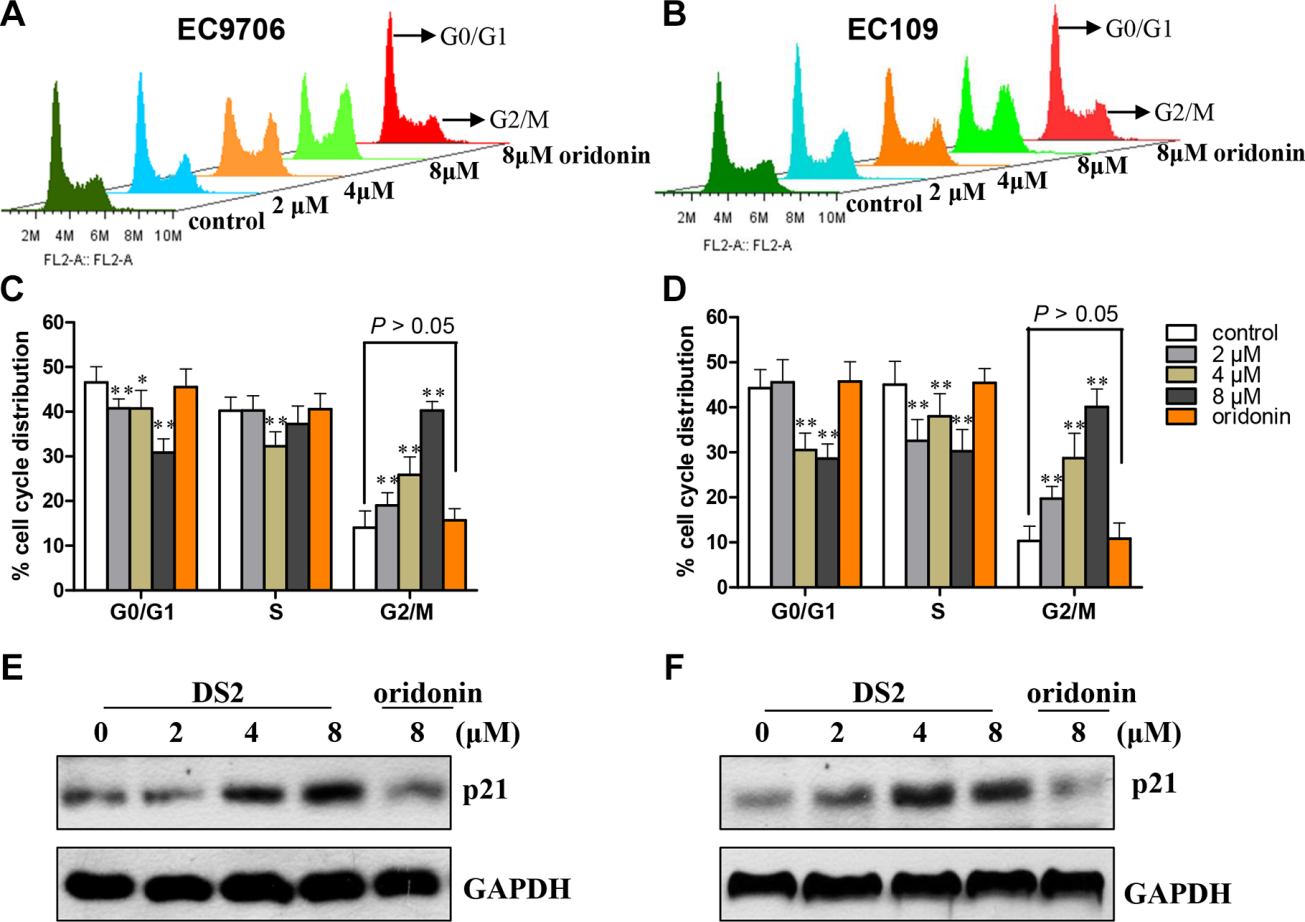
DS2 inhibits cell growth through G2/M phase arrest in ESCCs (**A**) and (**B**) Representative histograms depicting cell cycle distribution in EC9706 and EC109 cell cultures treated with 0.1% DMSO (control) or 2, 4, and 8 μM DS2 or 8 μM oridonin for 12 h. Similar results were observed in three independent experiments. (**C**) and (**D**) Data are presented as mean ± SD of triplicate samples. *P < 0.05; **P < 0.01 as compared with control. (**E**) and (**F**) After ESCC cells treated with 0.1% DMSO (control) or DS2 or oridonin at the indicated doses for 12 h, the protein levels of cell cycle regulatory molecule p21 were detected by western blot. The results were representatives of three independent experiments. GAPDH was used as loading control.

To identify whether DS2-induced anti-proliferation implicates changes in cell-cycle progression, cell cycle phase distribution was examined by us using flow cytometry. As shown in Figure 2, a dramatic increase of G2/M phase was observed in EC9706 and EC109 cells treated by DS2 at 2, 4 and 8 μM for 12 h, and a decrease in G0/G1 and S phase cells was simultaneously observed. (Figure 2A, 2B, 2C and 2D). Moreover, as a cyclin-dependent kinase inhibitor (CDKI), p21 protein levels were observably increased by DS2 in a dose-dependent manner (Figure 2E, 2F). However, all these alterations were not observed in both EC9706 and EC109 cells treated with 8 μM oridonin. These results showed that the antiproliferative activity of DS2 was related to G2/M phase cell cycle arrest.

### DS2 induces apoptosis of ESCC cells

**Figure 3:**
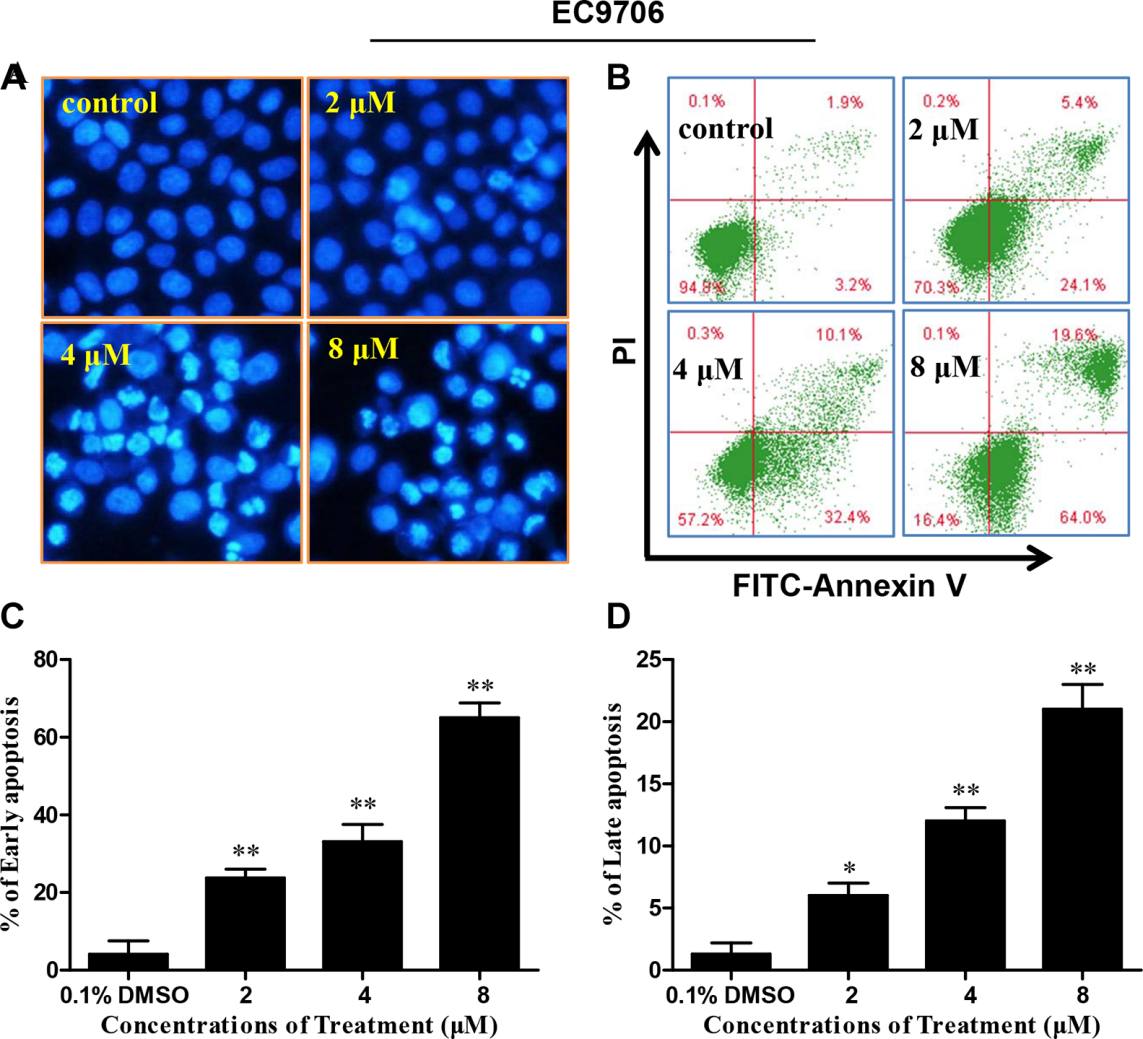
DS2 inhibits viability of EC9706 cells in association with apoptosis induction (**A**) Morphological changes in EC9706 cells treated with DS2 as indicated doses. After treatment with DS2 for 24 h, EC9706 cells were stained with Hoechst 33258 and observed under a fluorescence microscope (magnification 200×). (**B**) A representative picture of flow cytometry, and percentages of cells with apoptosis were given. After EC9706 cells were treated with 0.1% DMSO or DS2 at the indicated doses for 24 h, the cells were stained with FITC-Annexin V/PI and then analyzed by flow cytometry. (**C**) and (**D**) Statistical analysis of data obtained in flow cytometer. The experiments were repeated three times and the results were presented as mean ± SD. *P < 0.05 and **P < 0.01 as compared with control.

**Figure 4:**
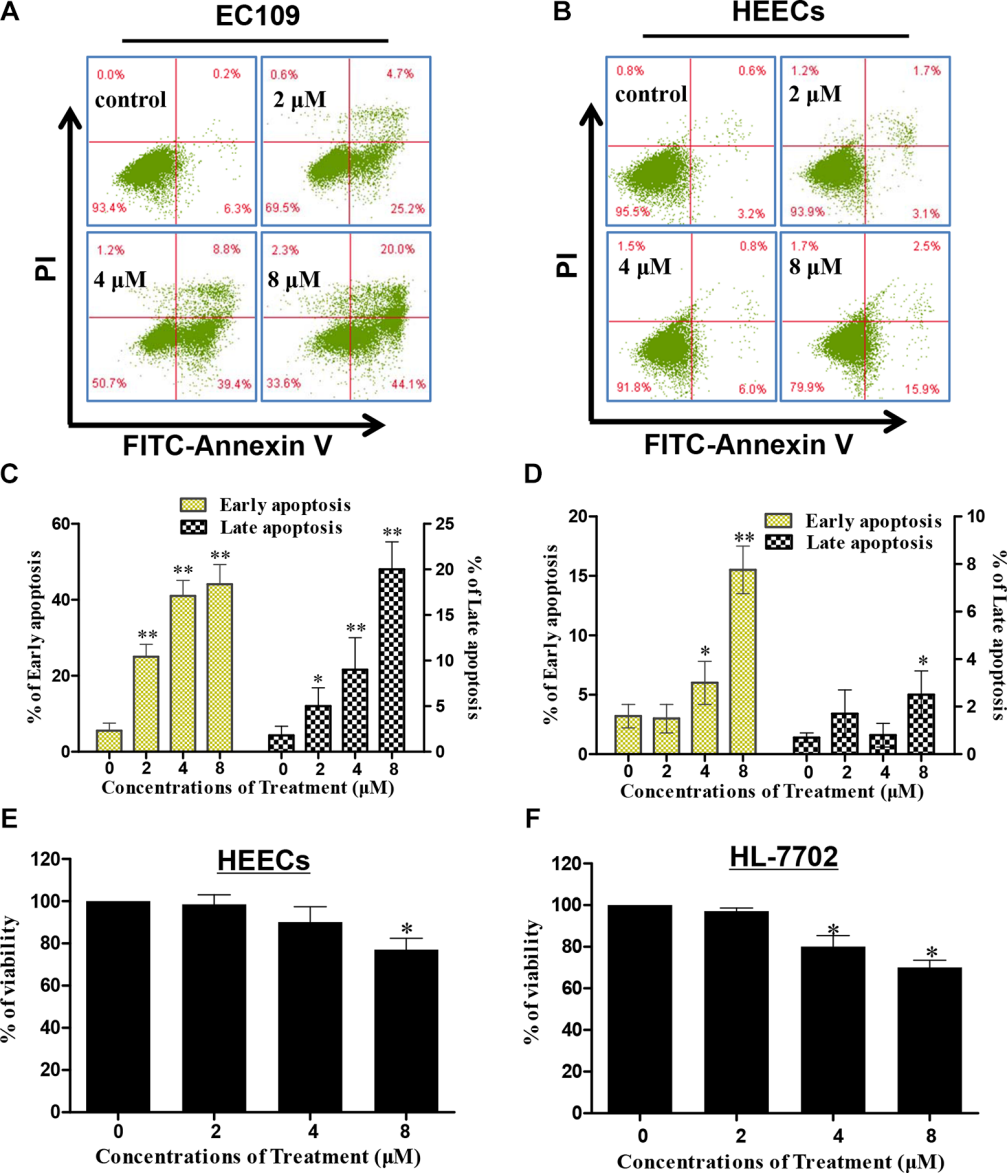
Effect of DS2 treatment for 24 h on EC109 cells, HEECs and normal human liver cells (HL-7702) (**A**) and (**B**) After EC109 cell and HEECs were treated with 0.1% DMSO or DS2 at the indicated doses for 24 h, the cells were stained with FITC-Annexin V/PI and then analyzed by flow cytometry. (**C**) and (**D**) Percentages of cells with early apoptosis or late apoptosis were summarized with histogram graphs. The experiments were repeated three times and the results were presented as mean ± SD. *P < 0.05 and ** *P* < 0.01 as compared with control. (**E**) and (**F**) Survival rates of HEECs and HL-7702 cells are determined by trypan blue dye exclusion assay. Data are presented as means ± SD of triplicate samples. *P < 0.05;**P < 0.01 as compared with control.

To determine whether the proliferation inhibitory effect of DS2 was also due to apoptosis, the EC9706 cells morphology was examined by the fluorescence microscopy stained with Hoechst 33258. As shown in the Figure 3A, cells treated by DS2 present typical apoptotic morphologies, such as cell crimp and rounding, as well as nuclear condensation and generation of apoptotic bodies, compared to controls. Similar phenomena were observed when EC109 cells were treated with DS2 (Figures not shown). The percentage of apoptotic cells with control and different doses of DS2 treatments was further determined by the flow cytometry analysis, following FITC-annexin V/PI staining. 2, 4 and 8 μM DS2 treatments of EC9706 cells for 24 hours resulted in a significant increment of FITC-Annexin V^+^/PI^−^ (early apoptosis) and FITC-Annexin V^+^/PI^+^ (late apoptosis) population, compared to untreated cells (Figure 3B, 3C and 3D). Similar result was obtained when EC109 cell line was treated with DS2 (Figure 4A and 4C). Interestingly, HEECs were significantly more resistant to DS2-induced apoptosis even at 8 μM concentration (Figure 4B and 4D). Furthermore, we also studied the sensitivities of HEECs and HL-7702 cells to DS2 by trypan blue dye exclusion assay, showed relatively insensitivity to DS2 as compared to those ESCC cell lines (Figure 4E and 4F). These results indicated that DS2 selectively caused apoptosis and proliferation inhibition in cancer cell lines without affecting normal cells significantly.

### DS2 results in MMP drop, cytochrome c release and cleavage of caspase-3/9 in EC9706 cells

**Figure 5:**
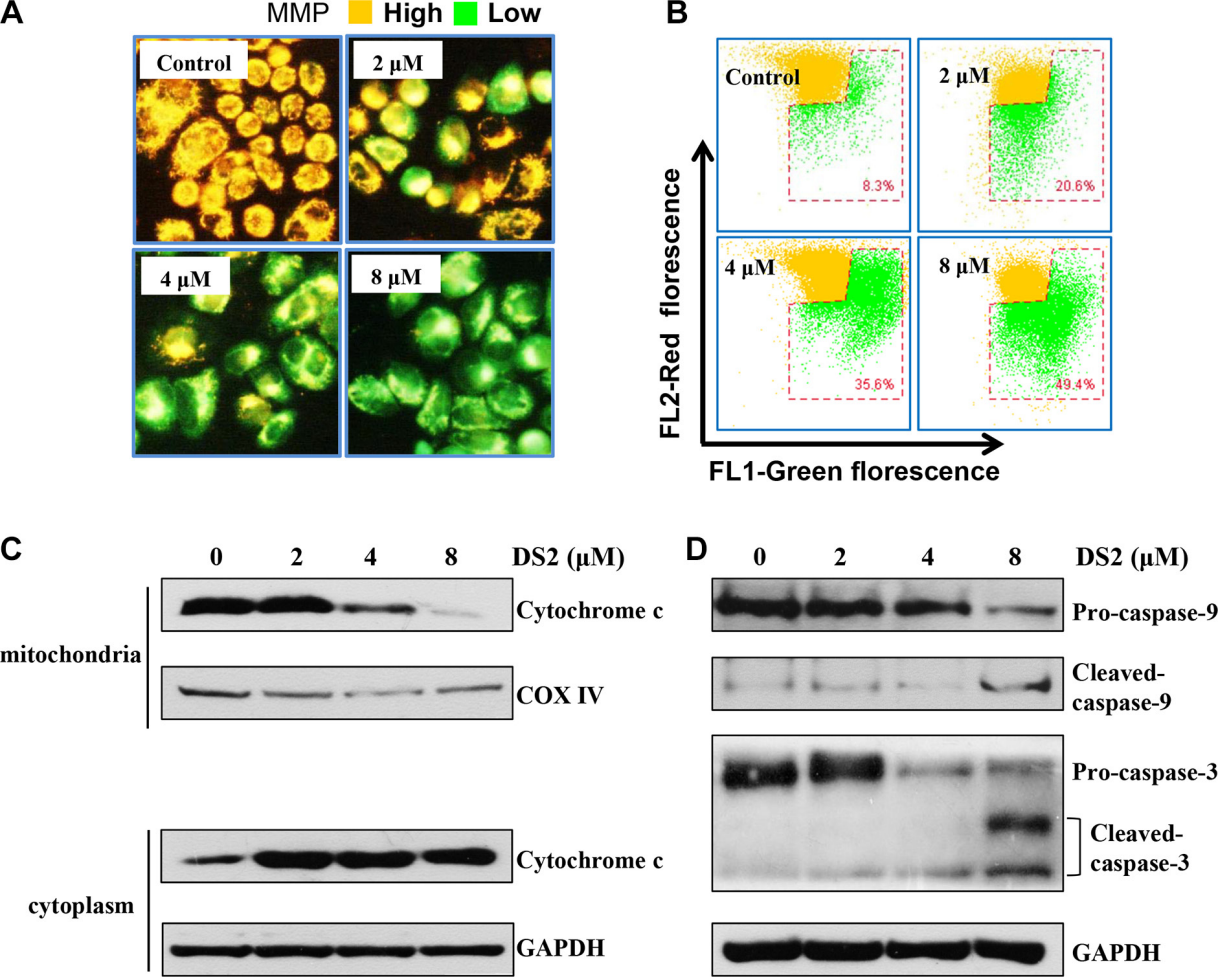
DS2 reduces the MMP and induces release of cytochrome c and the cleavage of caspase-9/3 EC9706 cells were incubated with DS2 at the indicated concentrations for 24 h, stained with JC-1 and then imaged by fluorescent microscope (**A**) and analyzed by flow cytometry, numbers in the bottom right gate, percentage of cells with low MMP (**B**). (**C**) Mitochondria and cytosolic extracts from indicated treatments for 24 h were prepared as described in Materials and Methods. Western blotting analysis was performed for cytochrome c levels. (**D**) Protein levels of cleaved caspase-9/-3 were determined by Western blot. GAPDH and COX IV were used as loading control.

On account of the MMP collapse is a key trigger for the mitochondria dependent apoptotic pathway [21], we next examined whether DS2 affected the MMP by the JC-1 staining. JC-1 is mitochondria selective and forms aggregates in normal mitochondria that leads to an orange fluorescence under exciting at 490 nm. However, in depolarized mitochondrial membranes, JC-1 forms monomeric and emits green fluorescence [22]. After EC9706 cells were treated with 2, 4 and 8 μM DS2 for 24 hours, a clear shift in JC-1 staining of the mitochondria from orange to green fluorescence was observed in a dose-dependent manner (Figure 5A). Figure 5B presents an increase in the percentage of cells in red gate, which represents cells with depolarized mitochondrial membranes. Compared with 8.3% in the control group, the number of cells with loss of MMP increased up to 20.6%, 35.6% and 49.4%, respectively, after the treatment with 2, 4 and 8 μM DS2 for 24 hours. Similar results were also observed in EC109 cells (Supplementary Figure S4). The loss of MMP will result in cytochrome c release from mitochondria to cytosol. Consistent with the above conclusions, a distinct cytochrome c release from mitochondria into cytosol in EC9706 cells in dose-dependent manner was observed (Figure 5C), leading to an increased expression of cleaved caspases 3/9 and a decreased expression of their proenzymes (Figure 5D).

### DS2 increases protein expressions of Bax in a concentration- and time-dependent manner

**Figure 6:**
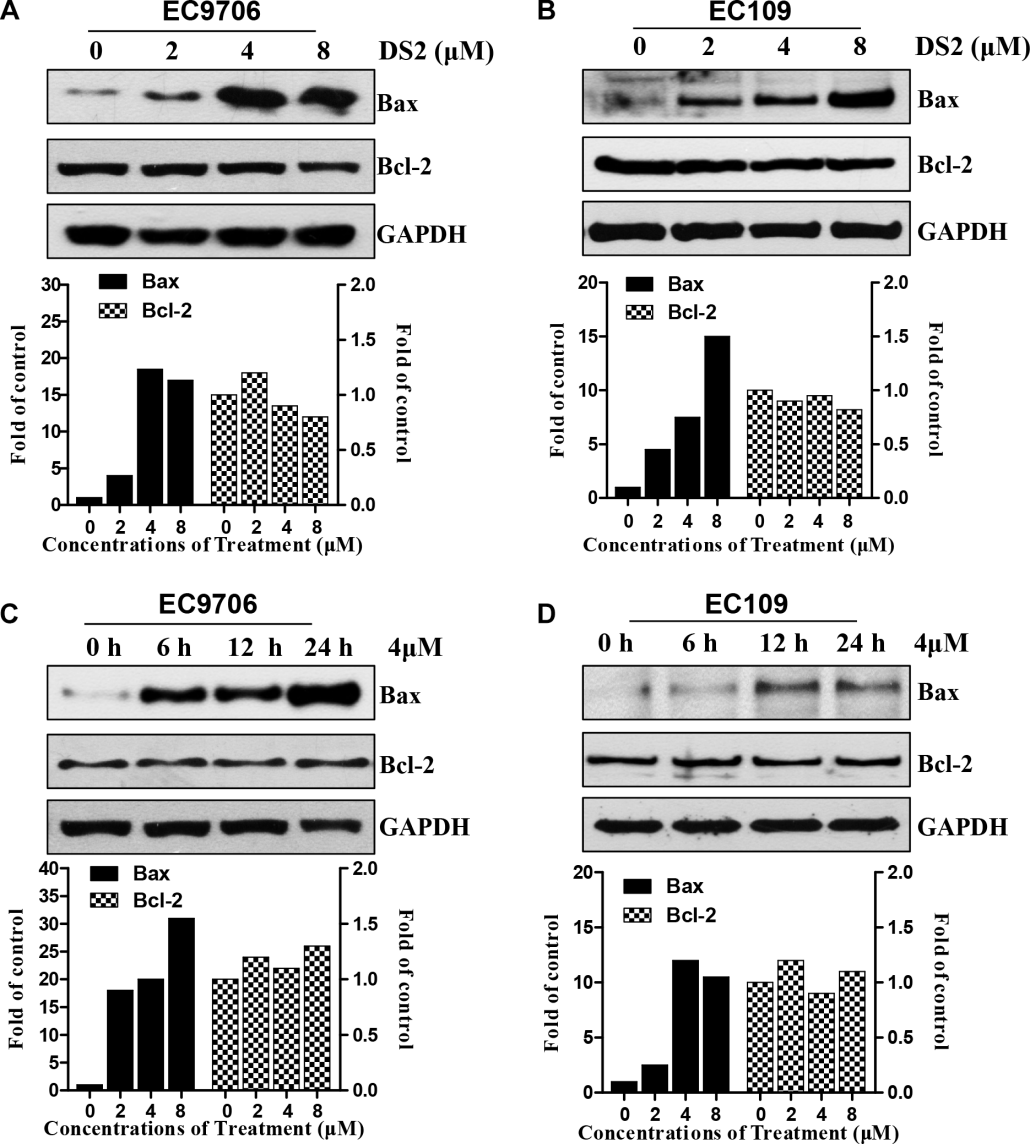
Effect of DS2 treatment on levels of Bax proteins Cells were treated with DS2 as indicated doses for 24 h or 4 μM DS2 for 6, 12, and 24 h, and then lysed in cell lysis buffer. (**A**) and (**B**) Immunoblotting for Bax using lysates from EC9706 or EC109 cells treated with the indicated doses. (**C**) and (**D**) Immunoblotting for Bax using lysates from EC9706 or EC109 cells treated with 4 μM DS2 for 6, 12, and 24 h. GAPDH was used as a loading control. Relative levels of Bax protein were quantified by densitometry measurement after adjusting levels of loading controls.

Because DS2 treatment disrupted MMP resulting in cytosolic release of cytochrome c in EC9706 cells, we subsequently tested whether the apoptosis induced by DS2 was regulated by Bax, a key proapoptotic molecule in the mitochondria-dependent apoptotic pathway [23]. Treatment of EC9706 and EC109 cells with the indicated concentrations DS2 resulted in a significant up-regulation of Bax protein in a concentration-dependent manner, compared with control (Figure 6A, and 6B). Moreover, treatment of EC9706 cells with 4 μM DS2 for 6, 12, and 24 hours caused a noticeable up-regulation of Bax expression by 18-, 20-, and 31-fold, respectively, compared with control (Figure 6C). The similar results were also observed in EC109 cells, and the induction of Bax protein by DS2 was obvious as early as 6 h after treatment and was sustained for the whole experimentation (Figure 6C and 6D). However, as shown in Figure 6, the level of Bcl-2, a key anti-apoptotic protein, did not alter in EC9706 and EC109 cells treated by DS2 in indicated concentrations and times. These results showed that the DS2-induced apoptosis in ESCC cells was probably attributable to the up-regulation of Bax.

### The loss of MMP and apoptosis caused by DS2 are associated with Bax

**Figure 7:**
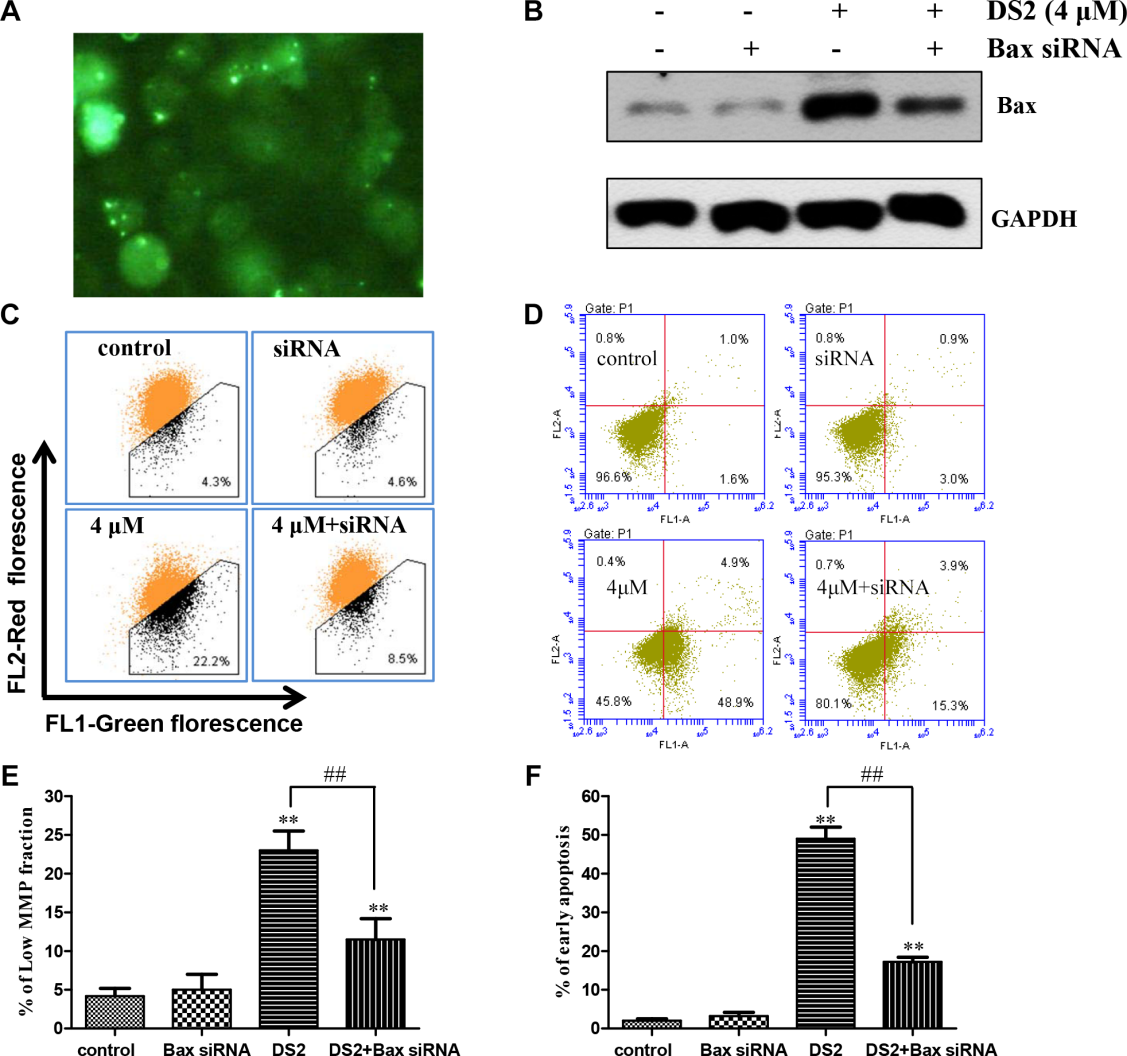
Bax siRNA conferred significant protection against DS2-induced MMP drop and apoptosis (**A**) Uptake of fluorescent-labeled siRNA in EC9706 cells. Cells were transfected with control fluorescent-labeled siRNA (green). Shown here are representative areas of cells. (**B**) EC9706 cells were treated with control, Bax siRNA, and then the cells were treated with 4 μM DS2 for 24 h. Total Bax levels were shown by representative immunoblots. (**C**) The low MMP fractions in EC9706 cells transiently transfected with a control siRNA or Bax siRNA and treated with either 0.1% DMSO (control) or 4 μM DS2 for 24 h. (**D**) Cell apoptosis in EC9706 cells transiently transfected with a control siRNA or Bax siRNA and treated with either 0.1% DMSO (control) or 4 μM DS2 for 24 h. (**E**) and (**F**) Statistical analysis of data about MMP and apoptosis obtained in flow cytometer. The experiments were repeated three times and the results were presented as mean ± SD. ^*^*P < 0.01 as compared with control; ^##^*P* < 0.01, significantly different between DS2-treated control siRNA and DS2-treated Bax siRNA groups by paired *t*-test.

To further investigate potential molecular mechanisms of DS2-induced apoptosis in EC cells, we hypothesized that Bax might play an important role in the regulation of DS2-induced apoptosis. Then we confirmed this hypothesis by using Bax siRNA. The transfection efficiency was determined by with a control fluorescent-labeled siRNA, and fluorescence was visualized using fluorescence microscope (green, Figure 7A). Furthermore, a representative immunoblot for Bax using lysates from control EC9706 cells and Bax siRNA-transfected cells following 12 h treatment with 4 μM DS2 is shown in Figure 7B. The expression of Bax protein was decrease in Bax siRNA-transfected cells even treatment with DS2. These results proved that the expression of Bax protein was silenced in Bax siRNA-transfected cells. The effect of DS2 treatment (4 μM, 12 h) on the loss of MMP and apoptosis were determined using Bax siRNA-transfected cells, and these results are shown in Figure 7C and 7D. The loss of MMP and apoptosis on treatment with DS2 was statistically significantly reversed in Bax siRNA-transfected cells (Figure 7E, 7F). Together, these results indicated that Bax was involved in DS2-induced cell death.

### DS2-induced apoptosis and growth inhibition in ESCC cell lines are due to ROS generation

**Figure 8:**
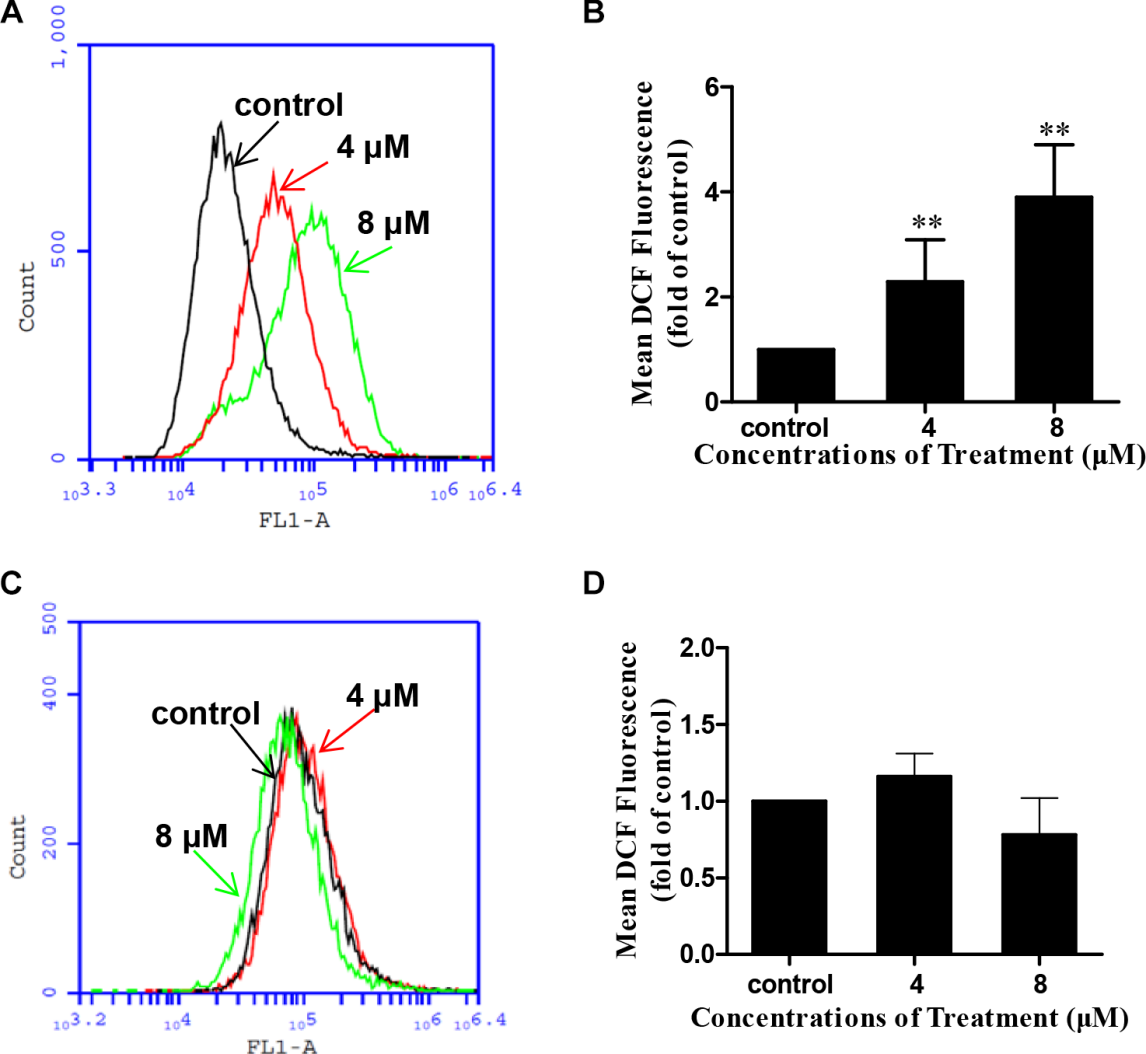
DS2 increased ROS generation in EC9706 cells EC9706 or HEEC cells were treated with DS2 at indicated doses for 8 h, followed by incubation with 10 μM DCFH-DA for 30 min at 37°C. The intracellular levels of ROS were determined by flow cytometer. (**A**) Representative percents of DCF positive cells analysed by flow cytometer in EC9706 cultures treated with 0.1% DMSO (control) or the indicated DS2 for 8 h. (**B**) Columns, mean DCF fluorescence in EC9706 cells of three independent experiments; bars, SD. **P < 0.01, DS2 versus control. (**C**) Representative percents of DCF positive cells analysed by flow cytometer in HEECs following 8 h exposure to DMSO or the indicated concentrations of DS2. (**D**) Statistical analysis of data about HEECs. The experiments were repeated three times and the results were presented as mean ± SD.

ROS is an upstream initiator for apoptosis and has been proved to be involved in the mitochondrial apoptotic pathway [24]. We have proved previously that Jaridonin-mediated apoptosis in ESCC cell lines correlates with ROS generation [9]. To investigate whether DS2 stimulates ROS generation in EC9706 cells, we measured intracellular ROS by flow cytometry method following staining with DCFH-DA [25]. The DS2-treated EC9706 and EC109 cells exhibited a concentration dependent enhancement in the percentage of DCF-positive cells (Figure 8A and Supplementary Figure S5). Similarly by flow cytometry analysis, 4 μΜ and 8 μΜ DS2 treatment for 8 hours statistically significant increased the mean DCF fluorescence up to about 2.3- and 3.9- fold, respectively, compared control treatment in EC9706 cells (*P* < 0.01) (Figure 8B). Interestingly, ROS generation was not observed in HEECs treated with similar DS2 for 8h (Figure 8C and 8D).

**Figure 9:**
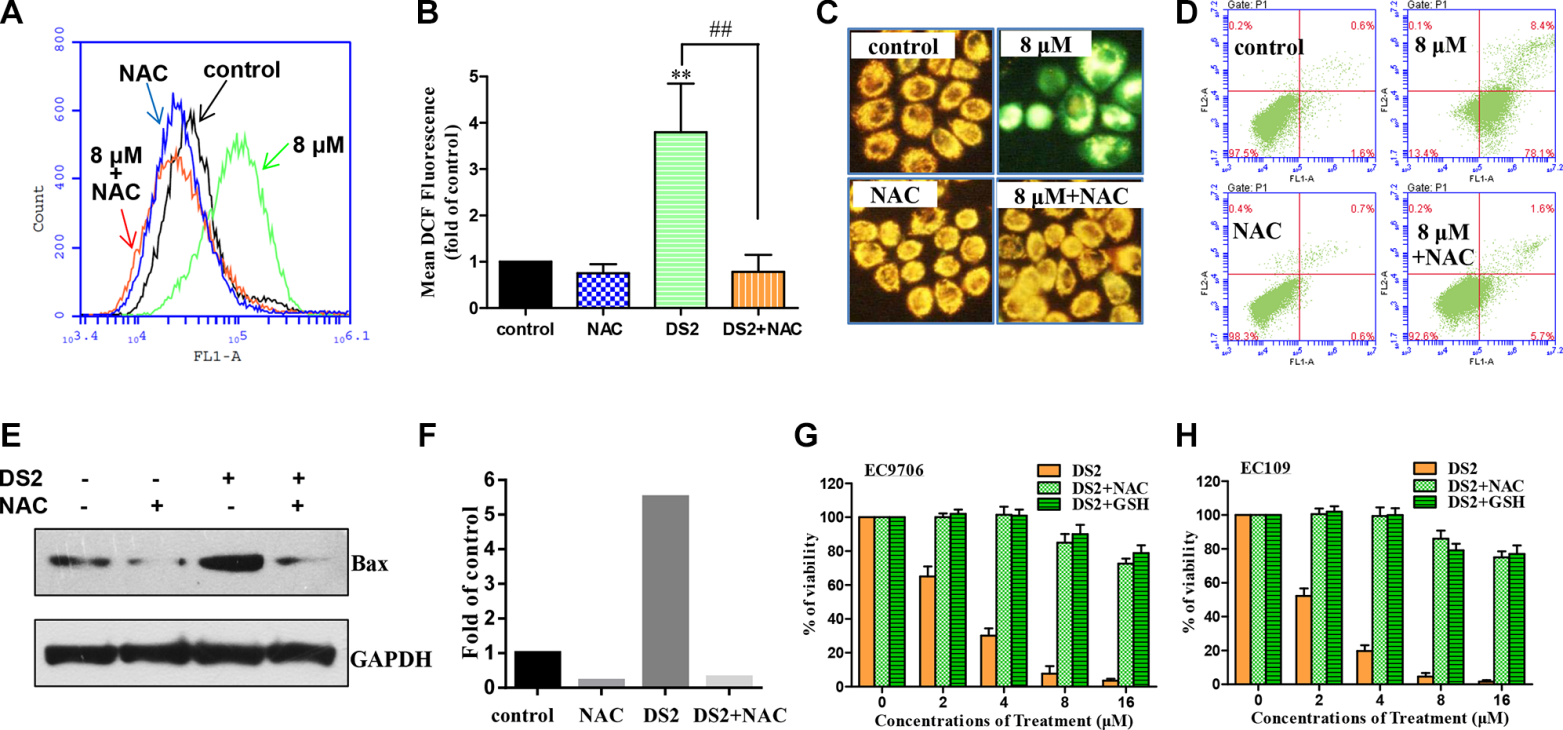
Effect of antioxidants on DS2 caused ROS generation, MMP drop, apoptosis, and Bax expression, as well as growth inhibition in ESCC cell lines (**A**) Flow cytometer analysis percentage of DCF-positive cells in EC9706 cells treated for 12 h with 0.1% DMSO (control) or 8 μM DS2 in the absence or presence of 5 mM NAC (2 h pretreatment). (**B**) Columns, mean DCF fluorescence of three independent experiments; bars, SD. **P < 0.01, DS2 versus control; ^##^*P* < 0.01, DS2 versus DS2+NAC. (**C**) EC9706 Cells were pretreated with or without 5 mM NAC, incubated with 8 μM DS2 for 24 h, stained with JC-1 and then imaged by fluorescent microscope. (**D**) Cell apoptosis in EC9706 cells following 24 h treatment with 0.1% DMSO (control) or 8 μM in the absence or presence of 5 mM NAC (2 h pretreatment). (**E**) Western blot analysis of Bax in EC9706 cells pretreated with or without 5 mM NAC for 2 h, and then incubated with 8 μM DS2 for 24 h. (**F**) Relative levels of Bax protein were quantified by densitometry measurement after adjusting levels of loading controls GAPDH. (**G**) and (**H**) EC9706 or EC109 Cells were pretreated with or without 5 mM NAC or 3 mM GSH for 2 h, and then incubated with the indicated doses of DS2 for 48 h. Cell viabilities were evaluated by MTT assay. Data were expressed as means ± SD. Each data point was an average of three independent experiments.

To further confirm this finding, we used 5 mM ROS scavenger NAC to pre-treat EC9706 and EC109 cells for 2 hours, followed by DS2 treatment. Figures 9A and 9B show that 8 μΜ DS2 induced ROS generation was completely attenuated in EC9706 cell. Moreover, NAC pretreatment conferred near-complete protection against DS2 induced drop of the MMP (Figure 9C and Supplementary Figure S6A) and apoptosis (Figure 9D and Supplementary Figure S6B), as well as up-regulation of Bax (Figure 9E and 9F). To strengthen the involvement of ROS in DS2-induced apoptosis, the effect on cell survival of two antioxidants NAC and GSH was investigated. Consistently, pretreatment with 5 mM L-NAC and 3 mM GSH significantly attenuated the inhibitory effect of DS2 on viabilities of EC9706 and EC109 cells (Figure 9G and 9H). Taken together, our data suggest that ROS production is required, at least in part, for DS2 inducing the mitochondria-dependent apoptotic pathway and inhibiting the growth of ECSS cells.

## DISCUSSION

Previous studies have indicated that natural ent-kaurane diterpenoids [26, 27] and its derivatives exhibit considerable anti-tumor activity [28, 29]. Especially, HAO472, an oridonin analog, was recently advanced into a phase I clinical trial (CTR20150246) in China by Hengrui Medicine Co. Ltd, for the treatment of acute myelogenous leukemia [30], which further confirms that ent-kaurane diterpenoids and their derivatives are the potential therapeutic agents in human cancers. In this study, we investigated DS2, a novel diterpenoid analog, the potential anti-proliferation activity using a few of human cancer cell lines, and found that DS2 displayed more potent anti-proliferation properties in a tumor-selective manner than oridonin. Compared with other cancer cells, ESCC cell lines EC9706 and EC109 were more sensitive to DS2 cytotoxicity, and this compound dramatically induced ESCC cell lines cycle arrest and apoptosis. Interestingly, these effects mediated by DS2 is not distinctly observed in normal tissue cells (eg. HEECs and HL-7702). Therefore, it is possible that this compound has a clinical translational potential for ESCC patients. Further exploration of the potential mechanisms of DS2-inducing apoptosis is indispensable.

To this end, we have traced from MMP loss and cytochrome c release to cleavage of caspase-9/3. Finally, we found that Bax proteins played a significant role in the DS2-induced apoptosis. Bax belongs to a major pro-apoptosis member of Bcl-2 family proteins, and is a central cell death regulator inducing mitochondrial membrane permeabilization and apoptosis-promoting molecules release from mitochondria to cytosol [31]. Many clinical anticancer drugs are known to induce Bax activation to facilitate apoptosis [32, 33], and increasing new molecular entities are synthesized and evaluated in direct activation of Bax [23, 34]. Intriguingly, accumulating evidence demonstrates that Bax can serve as a promising direct target for drug discovery [23]. Because DS2 treatment disrupted MMP leading to cytosolic release of cytochrome c in EC9706 cells, we then tested whether DS2-induced apoptosis was related to Bcl-2 family proteins. The present results indicated that the Bax protein levels were significantly increased by DS2 in a concentration- and time-dependent manner, and the DS2-mediated induction of Bax protein was obvious as early as 6 h after treatment and was sustained for the whole experimentation. But the change of Bcl-2 levels was not observed in EC9706 and EC109 cells. More interestingly, Bax knockdown in EC9706 cells could afford statistically significant protection against DS2-induced drop of MMP and apoptosis. These results pointed out that the DS2-induced apoptosis in ESCC cells was probably attributable to the up-regulation of Bax. It is important to point out, however, that Bax cannot fully explain the cytotoxicity caused by DS2 because the knockdown of Bax confers only partial protection against DS2-caused MMP collapse and apoptosis. Other regulators of mitochondria-mediated apoptosis, including promoters (Bak for example) or inhibitors (Bcl-xL for example), might be involved in DS2-induced apoptosis. Moreover, in ESCC cells, the parent compound, Jaridonin dramatically increases expression of p53, which is recognized as the upstream regulator of Bax [9]. Whether is p53 required for DS2, a Jaridonin analog induced cancer cells death? Therefore, further investigations are necessary to systematically identify these possibilities.

Cancer is frequently thought of as a cell cycle disease, cell cycle blockade is therefore considered as a valid strategy for wiping out cancer cells [35, 36]. In the present study, we also proved that DS2 worked on cell-cycle progression, and was more potential than oridonin. Previous studies have demonstrated that oridonin and its derivatives induced the expression of p21 and p21 dependent G2/M phase cell cycle arrest [37, 38]. Consistent with the previous reports, DS2 increased the levels of p21 protein in both the EC9706 and EC109 cells in dose-dependent manners. As a major endogenous CDKI, p21 has broad specificity for cyclin-dependent kinase (CDKs), and primarily prevents cell cycle progression. Besides, it also plays an important role in regulating other cellular processes, including apoptosis, senescence and differentiation [39]. It was reasonable to think that p21 was involved in DS2-induced G2/M phase arrest. Although the exact mechanisms remained to be elucidated, it was verified that DS2 inhibited proliferation via G2/M phase cell cycle arrest.

Elevated ROS levels and oxidative stress have been connected with apoptosis that was induced by a number of anticancer agents [40, 41]. Consistent with these reports, we demonstrated, for the first time, that the DS2-induced cell death in ESCC cells correlated with ROS generation. The present study indicates DS2 results in ROS generation in ESCC cells, and the DS2-induced drop of MMP, apoptosis, and increase of Bax are attenuated by NAC. Moreover, the growth inhibition caused by DS2 is also completely reversed on cotreatment with ROS scavenger, NAC and GSH in EC9706 and EC109 cells. It is possible that the level of ROS after treatment of cancer cells with DS2 is sufficient to provoke increase of Bax and apoptosis.

In conclusion, we have identified a novel apoptosis inducer and anticancer agent, DS2, a synthetic analog of natural ent-kaurane diterpene. The mechanisms of DS2–inducing apoptosis are, at least partly, through both ROS generation and the Bax-dependent mitochondria mediated pathway. Our findings should encourage not only the potential of DS2 as a candidate for the treatment of human ESCC but also the development of more hopeful diterpene derivatives for preventing and treating human cancers.

## MATERIALS AND METHODS

### Reagents and antibody

The primary antibodies for p21, cytochrome c, caspase-9, caspase-3, Bcl-2 and Bax were purchased from Santa Cruz Biotechnology, Inc. (Santa Cruz, CA). siRNA targeted against Bax and fluorescein conjugate siRNA were also from Santa Cruz Biotechnology. The siRNA transfection reagent Lipofectamine 2000 was from Invitrogen (Carlsbad, CA, USA). The antibody against GAPDH was from Good HERE Biotech Inc. (Hangzhou, China), and anti-COX IV antibody was from Abbkine Inc. (CA, USA). The horseradish peroxidase-conjugated secondary antibodies were obtained from Zhongshan Golden Bridge Biotech Inc. (Beijing, China). FITC-Annexin V/ PI apoptosis assays kit was from Biovision, Inc. (Palo Alto, CA). The ROS detection kit, GSH, NAC and JC-1 probe were all purchased from Beyotime Institute of Biotechnology (Jiangsu, China). Enhanced chemiluminescence (ECL) detection reagents were from Pierce Biotechnology, Inc. (Rockford, IL). Hoechst 33258, propidium iodide (PI) and 3-(4, 5-dimethylthiazol-2-yl)-2, 5-diphenyltetrazolium bromide (MTT) were from Sigma (St. Louis, USA).

### Cell culture conditions and compounds

Human esophageal cancer cell lines EC9706 and EC109, human gastric cancer cell line MGC-803, human prostate carcinoma cell line PC-3, and primary normal human liver cells (HL-7702) were purchased from China Center for Type Culture Collection (CCTCC, Shanghai, China). EC9706 cell line has been proven to be esophageal carcinoma of the fungating type, which is poorly-differentiated squamous cell carcinoma, and EC109 cell line is well-differentiated [19]. HEECs were obtained from Wuhan PriCells Biomedical Technology Co., Ltd. (Wuhan, China). All the cell lines were cultured in RPMI 1640 medium, containing 10% fetal bovine serum (FBS) and 1% penicillin/streptomycin. Cells were incubated at 37°C in a humidified atmosphere, containing 5% CO2. Pure oridonin was isolated from *Isodon rubescens*, and DS2 was synthesized in our laboratory. The chemical structures are shown in Figure 1A and were confirmed by NMR and IR data. Purities were determined by HPLC and were all above 98%. DS2 and oridonin were dissolved in dimethyl sulfoxide (DMSO), aliquoted, and stored at −80°C. The concentration of DMSO in culture medium was kept below 0.1% (v/v), a concentration known not to affect cell proliferation.

### Cell viability assay

MGC-803, PC-3, EC9706, and EC109 cells were plated at a density of 6 × 10^3^/well in 96-well plates in complete culture medium. After 24 hours, the cells were refreshed with fresh medium, untreated or treated as indicated in the figure legends. 24 h or 48 h later, MTT was added at a final concentration of 1 mg/mL and incubated at 37°C for 4 hours. The absorbance was determined at 490 nm. The effect of DS2 on HEECs and HL-7702 viability was determined by trypan blue dye exclusion assays as described previously [19].

### Cell cycle analysis

Cells were seeded in six-well plates and treated with DS2 at the indicated concentrations for 12 h, trypsinized, washed in PBS, and fixed in ice cold 70% ethanol overnight. The fixed cells were washed twice with PBS, and then resuspended in mixture solution (0.5% Triton X-100, 0.5 mg/ml RNase and 100 μg/ml PI in PBS), incubated at 37°C for 30 minutes. DNA contents/cells were analyzed in Accuri C6 flow cytometer (Becton, Dickinson & Co.; Franklin Lakes, NJ), with a total of 10,000 cells tested. The histograms of DNA distribution were modeled as a sum of G0/G1, S and G2/M phase, using FlowJo software.

### Hoechst 33258 staining and apoptosis analysis by flow cytometry

As we have described previously [9], the DS2-induced apoptosis was examined and identified according to the condensation and fragmentation of their nuclei by fluorescence microscopy after Hoechst 33258 staining. FITC- annexin V and PI was used to stain and identify subpopulations of cells with membrane changes (early stage apoptosis) and the associated loss of membrane integrity (necrotic or late apoptotic cells), respectively.

### Detection of MMP

MMP was measured using a potential-sensitive dye JC-1 [42]. Briefly, cells were exposed to DS2 alone at the indicated concentrations or DS2 in combination with NAC (5 mM) for 24 h. At the end of the incubation period, the cells were collected and washed twice with cold PBS, and then incubated with medium containing 10 μg/mL JC-1 for 30 min at 37°C. The MMP was measured under an excitation at 488 nm using a fluorescence microscopy, or analyzed by flow cytometry.

### Determination of cytochrome c release from the mitochondria

EC9706 cells were treated with 2, 4 and 8 μM DS2 for 24 hours. The mitochondria and cytosol fractions were prepared as described before [9]. Briefly, the homogenate was subjected to centrifuging at 600 g for 10 minutes to remove nuclei and unbroken cells. Then the supernatant was collected and centrifuged again at 12,000 g for 30 minutes at 4°C to obtain the cytosol (supernatant) and the mitochondria (pellet) fraction. Samples of the cytosol and the mitochondria were dissolved in lyses buffer were subjected to western blotting.

### Western blotting analysis

After treatment under each experimental condition, cells were lysed as described previously. Clarified protein lysates (30–80 μg) were electrophoretically resolved by 10% SDS-polyacrylamide gel, transferred to nitrocellulose membranes, and probed with primary antibodies. Immunoblotting was performed as described by us previously [9].

### Measurement of ROS generation

Intracellular ROS generation was determined using the redox-sensitive probes 2′, 7′-dichlorodihydrofluorescein diacetate (DCFH-DA) [43]. Control and treated cells as indicated in the figure legends were washed twice with PBS, and then incubated with serum free medium containing 10 μM DCFH-DA for 30 minutes at 37°C. Cells were washed three times with serum free RPMI1640 medium to remove extracellular fluorescent dye. The fluorescence signal was determined by a flow cytometer.

### siRNA transfection

EC9706 were transfected with Bax siRNA, control siRNA, fluorescein conjugate control siRNA as per the manufacturer's instruction with minor modifications. For transfection, cells were plated in 6-well plates and transfected at 30% confluence with siRNA duplexes (final concentration 70 nM) using Oligofectamine according to the manufacturer's recommendations. After 48 h, cells were treated with 4 μM DS2 or 0.1% DMSO for 12 h. Both floating and adherent cells were collected, washed with PBS, and processed for MMP and apoptosis analysis as described above.

### Statistics

Data are expressed as mean ± standard deviation (SD). The significance of two groups was determined with a two-tailed student's *t*-test. Analysis of multiple groups was performed by analysis of variance (ANOVA). The differences were considered significant at *P* < 0.05.

## SUPPLEMENTARY FIGURES


