# Supplementary material for: Induction of the mitochondria-mediated apoptosis in human esophageal cancer cells by DS2, a newly synthetic diterpenoid analog, is regulated by Bax and caused by generation of reactive oxygen species
Source: Oncotarget. 2016 Nov 11;7(52):86211–24. doi: 10.18632/oncotarget.13367 (PMC5349908; doi:10.18632/oncotarget.13367)
Supplement: Supplementary file 1 [file oncotarget-07-86211-s001.pdf]

Induction of the mitochondria-mediated apoptosis in human esophageal cancer cells by DS2, a newly synthetic diterpenoid analog, is regulated by Bax and caused by generation of reactive oxygen species

Supplementary Materials

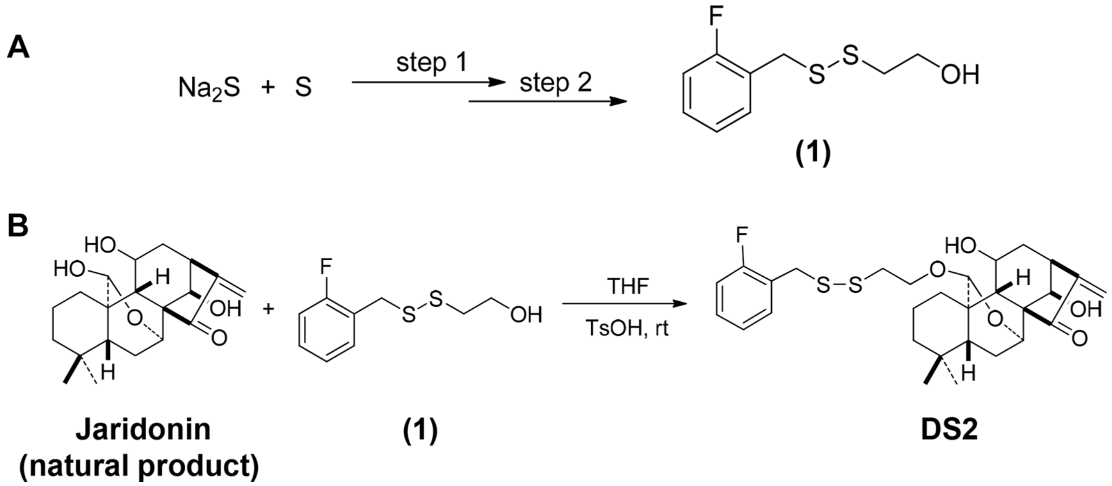

Supplementary Figure S1: Synthesis of DS2. (**A**) Synthesis of intermediates containing disulfide bond. Reagents and conditions of step 1 : H2O, 50°C, 30 min; Reagents and conditions of step 2: CHCl3, C2H5OBr, Tetrabutylammonium bromide (TBAB), 2-fluorobenzyl chloride, 50°C; (1) Intermediates containing disulfide bond. (**B**) Synthesis of the target compound DS2. Jaridonin is a new natural ent-kaurene diterpenoid, isolated from *Isodon rubescens* by us firstly.

DS2: white solid; Yield: 70%; **IR** (KBr, cm^{-1}): 3423, 2928, 2866, 1723, 1644, 1490, 1363, 1232, 1101, 1016, 952, 757; **^{1}H NMR** (400 MHz, $CDCl_3$) δppm: 7.82 (s, 1H, H-27), 7.69 (d, *J* = 7.9 Hz, 1H, H-28), 7.44 (t, *J* = 7.9 Hz, 1H, H-25), 7.38 (d, *J* = 7.7 Hz, 1H, H-26), 6.02, 5.30 (s, each 1H, H-17), 5.17 (s, 1H, H-20), 4.86 (d, *J* = 7 Hz, 1H, H-14-OH), 4.74 (t, *J* = 9.0 Hz, 1H, H-14α), 4.15 (dt, *J* = 15.4, 7.7 Hz, 1H, H-11α), 4.22 – 3.99 (m, 3H, H-11-OH, H-21a, H-7β), 3.77 (m, 1H, H-21b), 3.57 – 3.41 (m, 2H, H-23) 3.11 – 2.96 (m, 2H, H-6β, H-12α), 2.75 (m, 2H, H-22), 2.40 – 2.22 (m, 1H, H-1α), 2.07 (s, 1H, H-13α), 1.75 (d, *J* = 39.0 Hz, 1H, H-6α), 1.45 (s, 1H, H-3α), 1.50 – 1.31 (m, 6H, H-2, H-1β, H-12β, H-5β, H-9β), 1.17 (m, 1H, H-3β), 0.91 (s, 3H, H-19), 0.87 (s, 3H, H-18); **^{13}C NMR** ($CDCl_3$) δppm: 205.30 (C-15), 159.76 (C-29), 150.88 (C-16), 131.41 (C-25), 130.97 (C-27), 129.46 (C-24), 128.95 (C-26), 117.51 (C-17), 115.53 (C-28), 100.49 (C-20), 71.34 (C-14), 66.48 (C-7), 66.21 (C-21), 65.15 (C-11), 57.93 (C-8), 56.69 (C-9), 48.36 (C-5), 42.16 (C-13), 41.42 (C-12), 40.51 (C-3), 39.98 (C-10), 38.68 (C-22), 36.49 (C-23), 33.97 (C-4), 32.79 (C-18), 30.36 (C-1), 24.53 (C-6), 20.93 (C-19), 18.24 (C-2).

Supplementary Figure S2: Data of IR, ^{1}H NMR, and ^{13}C NMR.

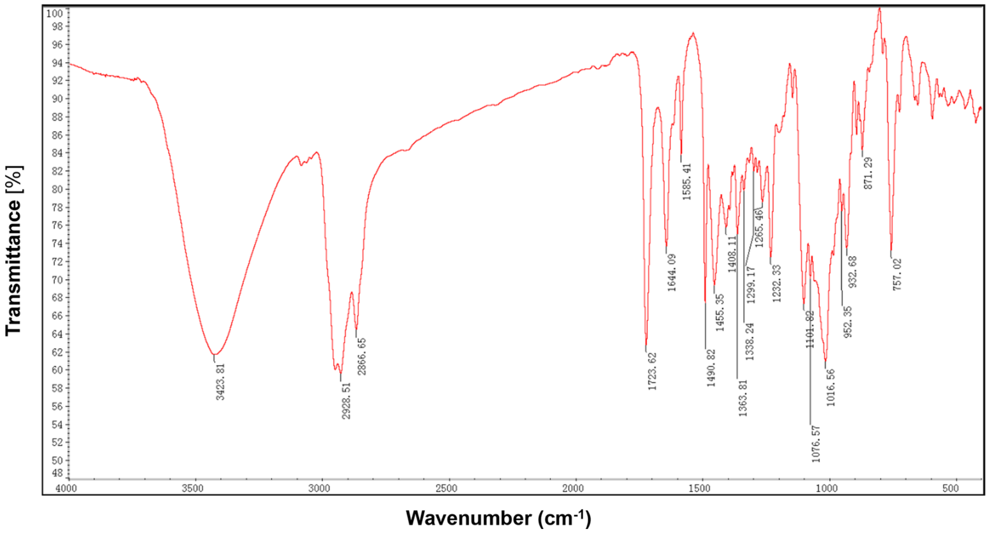

Supplementary Figure S3: Infrared spectroscopy (IR) of DS2.

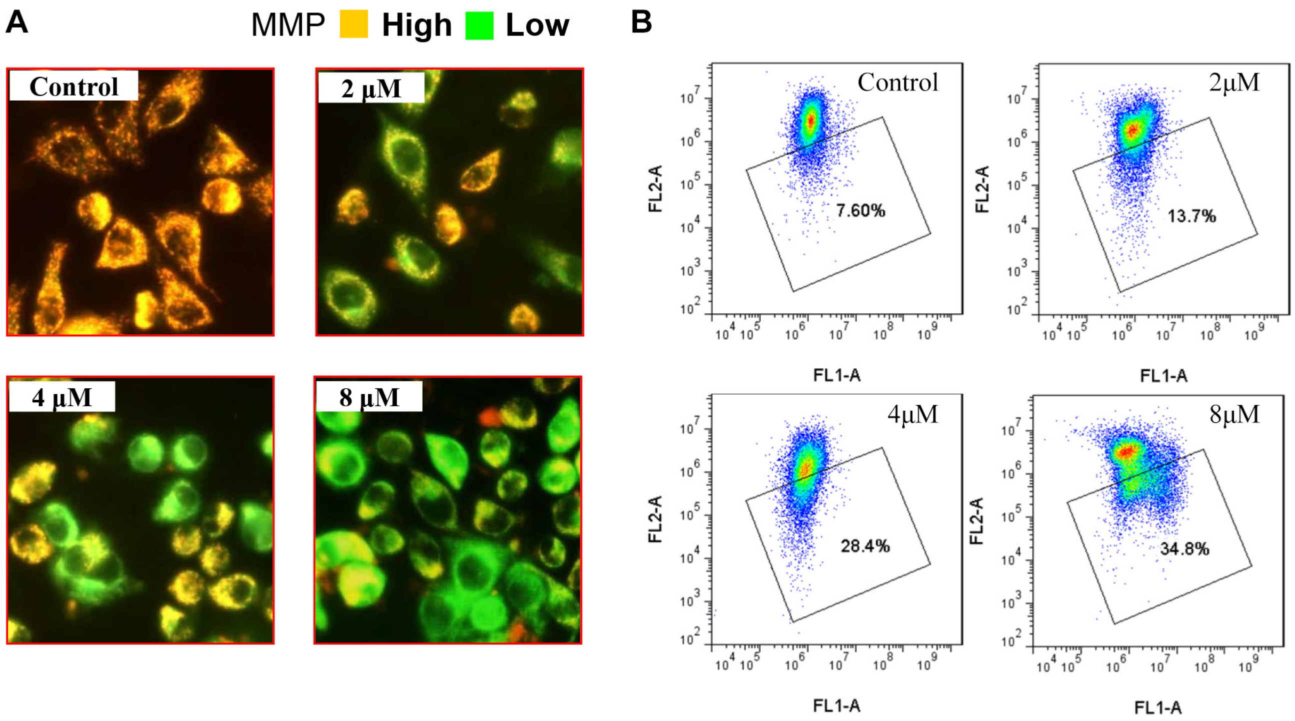

Supplementary Figure S4: DS2 induces the drop of MMP in EC109. EC109 cells were incubated with DS2 at the indicated concentrations for 24 h, stained with JC-1 and then imaged by fluorescent microscope (**A**) and analyzed by flow cytometry (magnification 200×), numbers in the bottom right gate, percentage of cells with low MMP (**B**).

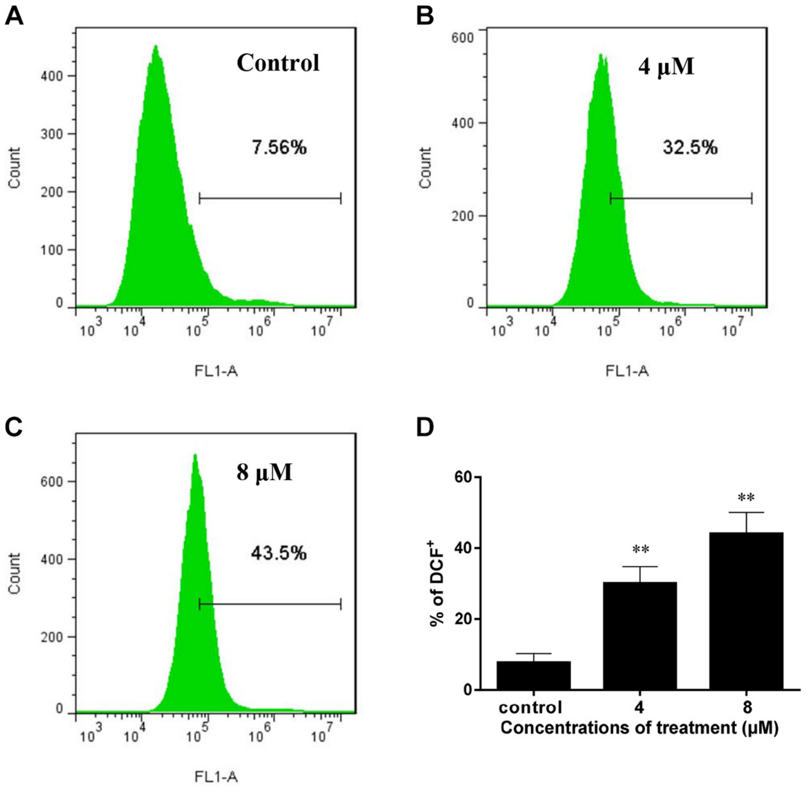

Supplementary Figure S5: DS2 induced ROS generation in EC109 cells. EC109 cells were treated with DS2 at indicated doses for 8 h, followed by incubation with 10 μM DCFH-DA for 30 min at 37°C. The intracellular levels of ROS were determined by flow cytometer. (**A**, **B** and **C**) Representative percentages of DCF positive cells were analyzed by flow cytometry. (**D**) Bar graphs for percentages of cell producing ROS. Columns, means of three independent experiments. $^{**}P < 0.01$ for DS2 versus control.

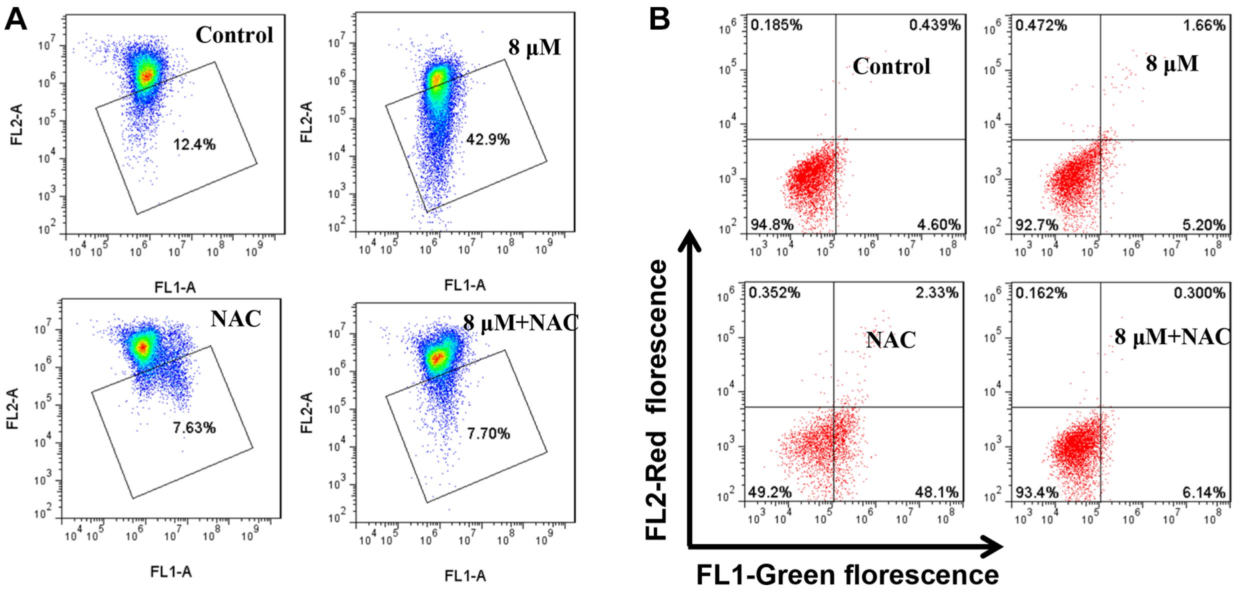

Supplementary Figure S6: The effect of DS2 on the MMP and cell apoptosis were reversed by antioxidant in EC109. EC109 cells were pretreated with or without 5 mM NAC for 2 hours, and then incubated with DS2 for additional 24 hours. (**A**) The cells were stained with JC-1 and analyzed by flow cytometry, and the numbers in the bottom right gate are percentage of cells with low MMP. (**B**) EC109 cells were treated with 0.1% DMSO (control) or 8 μM for 24 h in the absence or presence of 5 mM NAC, and the cell apoptosis was analyzed by flow cytometry.
